# A 6-Week Web-Based Osteoarthritis Treatment Program: Observational Quasi-Experimental Study

**DOI:** 10.2196/jmir.9255

**Published:** 2017-12-18

**Authors:** Håkan Nero, Jakob Dahlberg, Leif E Dahlberg

**Affiliations:** ^1^ Orthopaedics, Department of Clinical Sciences Lund Faculty of Medicine Lund University Lund Sweden; ^2^ Arthro Therapeutics AB Malmö Sweden; ^3^ Orthopaedics, Department of Clinical Sciences Lund Skåne University Hospital Lund University Lund Sweden

**Keywords:** digital treatment, eHealth, telemedicine, osteoarthritis, pain, physical function

## Abstract

**Background:**

Osteoarthritis (OA) is one of the most common causes of disability, with a burden of disease estimated to increase over time. Joint Academy, a Web-based treatment for individuals with clinically verified knee or hip OA, was developed to increase access to and facilitate implementation of evidence-based nonsurgical OA treatment in accordance with international guidelines.

**Objective:**

The primary aim of this study was to evaluate joint pain, physical function, and health-related quality of life (HRQoL) over time of users of the Joint Academy program.

**Methods:**

We enrolled 350 patients who were recruited online and completed the initial health journal in the 6-week program. We asked patients to complete an eHealth journal and e-questionnaires, including pain level assessed by a numerical rating scale, as well as a physical function evaluation using the 30-second chair-stand test. In addition, we assessed HRQoL using the 3-level version of the EQ-5D. We also asked participants whether they experienced difficulty walking and were afraid of physical activity due to their OA and their desire for surgery. We collected descriptive data and compared pre- versus postintervention data. As a reference group, we included results retrieved from the Swedish well-structured face-to-face self-supportive OA management program Better Management of Patients With Osteoarthritis (BOA).

**Results:**

Of the study cohort (n=350 patients; 239 women, mean age 62 years, mean body mass index 27 kg/m2), 71.4% (n=250) completed the program and were included in the study. We used the questionnaires to secure a clinical diagnosis of OA and to establish baseline study values. After 6 weeks of treatment, the change in mean numerical rating scale was larger than the minimal clinical difference (5.4 vs 4.1; *P*<.001), while physical function increased (from 10.88 to 13.14; *P*<.001). The percentage of participants having walking difficulties decreased from 81.7% (196/240) to 62.1% (149/240; *P*<.001), those afraid of being physically active decreased from 22.1% (53/240) to 6.7% (16/240; *P*<.001), and 22.0% (55/250) reported that they had reduced the amount of OA-related medication. After 6 weeks, 24% (13/54) of those desiring surgery at the start of the program were no longer interested. In addition, the comparison between Joint Academy and the BOA program showed similar levels of pain at 3 months, but suggested greater reduction with the use of Joint Academy due to a higher level of pain at baseline.

**Conclusions:**

The reported data suggest that participation in Joint Academy is associated with a clinically relevant decrease in pain and an increase in physical function and HRQoL, as well as a decreasing fear of physical activity. This innovative Web-based OA treatment is scalable, is population specific, and can reach a large number of individuals with impaired joints who have Internet access.

## Introduction

Chronic conditions, at accelerating rates globally, are the leading causes of death and disability [1]. Due to an increase in average life expectancy and a higher prevalence of obesity and sports-related joint injuries, the estimated disease burden of one of the most common and costly disabling chronic diseases, osteoarthritis (OA), will markedly increase over time [2]. Thus, health care systems need to prepare for the large increase in demand for OA treatment [3]. According to evidence and international guidelines, the primary treatment of knee and hip OA is nonsurgical and based on exercise, information, and, when necessary, weight loss [4-6]. Despite these evidence-based guidelines, only a minority of all people with OA receive adequate treatment; for instance, more than 50% of patients with OA in Sweden are referred to surgery as a primary option [7].

An alternative to nonsurgical treatment delivered face-to-face to individuals with OA is eHealth. Web-based treatment can increase the accessibility to care, due to its inherent flexibility in comparison with face-to-face treatment. In addition, there are numerous examples of Web-based programs in the literature, reporting beneficial effects on key health-related outcomes [8-11]. To this end, we developed the Web-based OA treatment platform Joint Academy [12] based on national and international guidelines and on a successful Swedish face-to-face concept (Better Management of Patients With Osteoarthritis [BOA] program), in which, to date, more than 90,000 patients have participated [7]. Joint Academy entails a 6-week Web-based program that adheres to evidence-based OA treatment. According to a pilot study, the results of the Joint Academy program on joint pain in individuals with knee or hip OA were promising [12]. Yet the level of physical function and health-related quality of life (HRQoL) after an eHealth intervention are unknown in a larger population. Hence, the primary aim of this study was to evaluate joint pain, physical function, and HRQoL in users of the Joint Academy. The secondary aims were to investigate whether use of the 6-week program is associated with decrease fear of physical activity and desire for surgery, and improve self-reported difficulties walking. We compared pain results with those in the structured face-to-face BOA program.

## Methods

This was an observational and quasi-experimental study. We recruited patients via advertisements and campaigns on the Web to participate in Joint Academy, a 6-week Web-based OA treatment program that provides information, exercises, an online physiotherapist, and education regarding factors of relevance to OA, including lifestyle. The advertisements were placed on search engines and social networks. The program is accessed by using a smartphone, tablet, or computer and encourages user activity by sending email prompts to participants on a regular basis, as described in detail previously [12]. An orthopedic surgeon and a physiotherapist, using synchronous and asynchronous chat conversations, supervised the clinical progress and were responsible for making a correct patient diagnosis and for identifying eligible patients. The program contained 2 telephone consultations with a physiotherapist that were compulsory, 1 at the start and 1 after 6 weeks. Participants’ costs were covered through either a private or a public health plan.

We asked patients to complete an e-questionnaire regarding their overall health and OA symptoms. The e-questionnaire also assessed pain level using the sensitive and reliable numerical rating scale [13], HRQoL using the 3-level EQ-5D (EQ-5D-3L) [14], and physical function measured by the 30-second chair-stand test [15], where participants sit and stand from a chair for 30 seconds, and the number of repetitions is recorded. Participants were also asked whether they experienced difficulty walking and were afraid of physical activity due to their OA (dichotomous replies), as well as whether they desired surgery. All outcomes were self-assessed and chosen according to the International Consortium for Health Outcomes Measurement Standard Set for Hip & Knee Osteoarthritis [16]. To enable a comparison with face-to-face treatment, we matched data from the Joint Academy database on reported joint pain with results at 12 weeks from the BOA initiative, found in the BOA yearly report of 2015 [17].

We defined as completers those participants who reported one of the main outcomes (joint pain, HRQoL, or physical function) at baseline and postintervention in the hip or knee. We excluded outliers of adherence—that is, participants with an activity level in the program below 10%.

Statistical analysis of pre- versus postintervention measurements was by 2-tailed *t* test for pain level, physical function, and HRQoL. For dichotomous and polytomous variables, we used McNemar and Madansky test of marginal homogeneity, respectively. The calculations were performed using the Statsmodels package in Python v3.6.1 (Python Software Foundation) and the coin library in R v3.4.1 (R Foundation).

We collected consent to participate at registration and obtained ethical approval from the Regional Board of Ethics in Lund, Sweden (Dnr 2017/651).

The datasets for this study are available from the corresponding author upon reasonable request.

## Results

The study cohort comprised 350 individuals with a clinical diagnosis of OA in accordance with American College of Rheumatology criteria and international guidelines as judged by a physiotherapist or an orthopedic surgeon after scrutinizing the questionnaires [18]. Table 1 presents patient demographics.

The majority reported “working” as their occupational status, while 4.0% (14/350) were on sick leave. Most participants reported level of physical activity of 90 to 150 (85/350, 24.3%) and structured exercise of less than 30 minutes per week (97/350, 27.7%).

On the basis of the requirements of available data and level of adherence, we included 71.4% (n=250) of the study cohort in the study. We used the questionnaires within the program to secure a clinical diagnosis of OA and to establish baseline study values. Table 2 shows the changes in the outcomes studied. Data were missing for pain measurement, HRQoL, and physical function; however, none exceeded 10% (10/250, 4.0%; 15/250, 6.0%; and 16/250, 6.4%, respectively).

**Figure 1:**
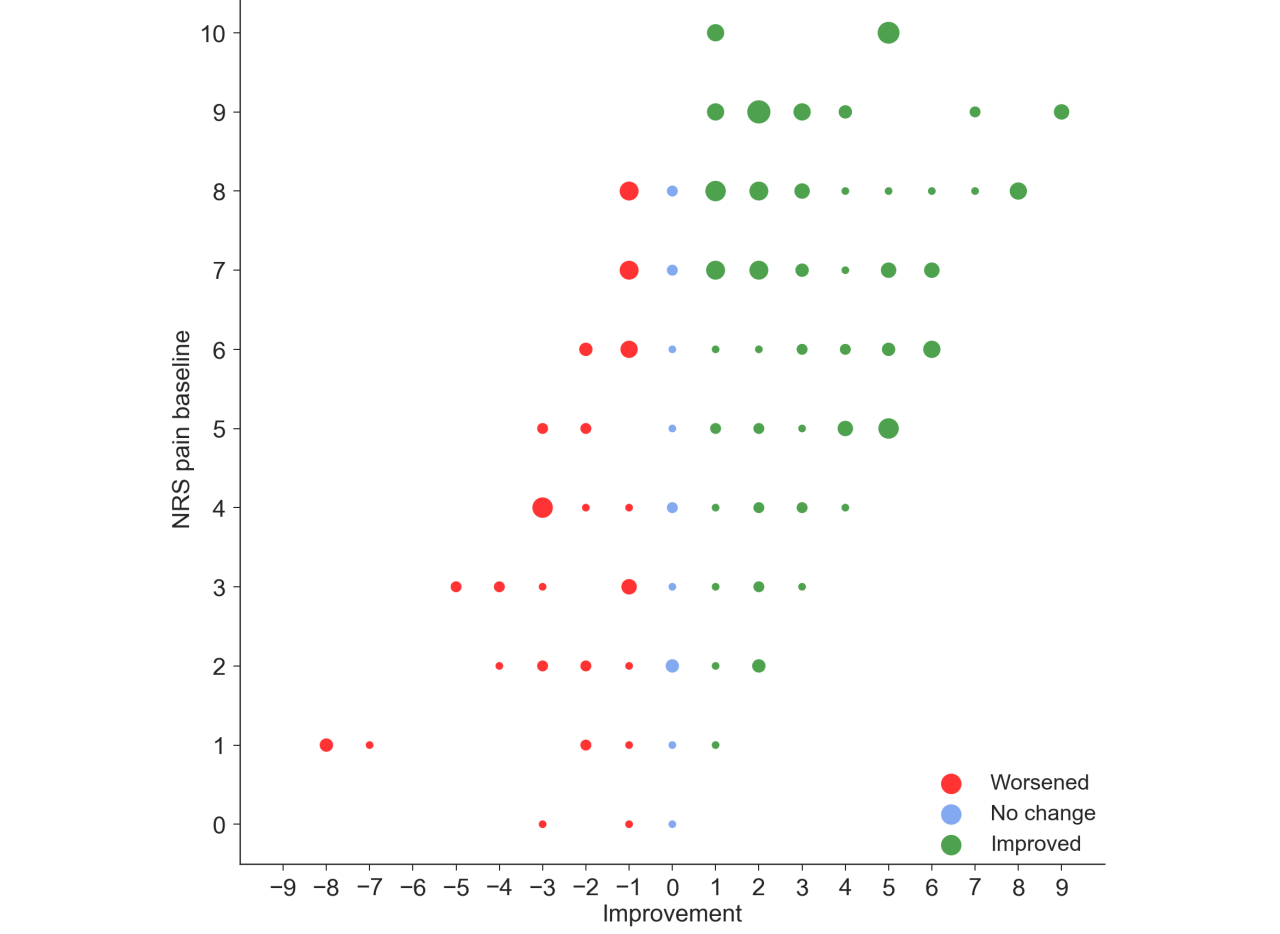
Scatterplot of pain improvement related to baseline joint pain. Plot size is dependent on number of participants at each point; larger plots denote higher number of participants. NRS: numerical rating scale (score range 0-10).

When investigating the distribution of pain improvement over self-reported joint pain at baseline, we observed improvement in patients with both low and high initial pain. However, most of those reporting improvement had more severe pain at baseline (Figure 1).

**Table 1:** Patient characteristics at baseline (N=350).

Characteristic		Count
Age (years), mean (SD^a^)		62 (10)
Body mass index (kg/m^2^), mean (SD)		27 (5)
Female, n (%)		239 (68.3)
**Reported pain locations, n (%)**			
	Knee	201 (57.4)
	Hip	145 (41.4)
	Other	4 (1.1)
Previous OA^b^ treatment, n (%)		61 (17.4)
OA medication use^c^, n (%)		168 (48.0)
**Occupational status, n**			
	Working	176
	Retired	149
	Other	25
Previous surgery^d^, n (%)		45 (12.9)
Previous diagnosis^e^, n (%)		289^f^ (86.8)

^a^SD: standard deviation.

^b^OA: osteoarthritis.

^c^Patients taking OA-related medication during the last 6 months.

^d^Patients who had joint surgery before entering the program.

^e^Patients with a diagnosis before entering program.

^f^Number of patients reporting outcome, n=333

**Table 2:** Outcome measures at baseline and at follow-up (n=250).

Metric	No. of patients reporting outcome	Baseline	Follow-up (after 6 weeks)	*P* value
NRS^a^pain score, mean (SD)	240	5.4 (2.2)	4.1 (2.4)	<.001
EQ-5D-3L^b^score, mean (SD)	235	0.65 (0.14)	0.69 (0.15)	.002
Functionality^c^, mean (SD)	234	10.88 (4.50)	13.14 (4.74)	<.001
Difficulty walking^d^, n (%)	240	196 (81.7)	149 (62.1)	<.001
Afraid of activity^d^, n (%)	240	53 (22.1)	16 (6.7)	<.001

^a^Numerical rating scale, score range 0-10.

^b^3-level EQ-5D.

^c^Physical function measured as the number of repetitions in the 30-second chair-stand test.

^d^Dichotomous response (yes/no).

**Table 3:** Patient-rated mean (SD) joint pain with use of Joint Academy and BOA at different time points.

Time points	Joint Academy^a^	BOA^b^
Baseline	5.4 (2.2)	48 (N/A^c^)
3 months	3.5 (2.5)	37 (N/A)

^a^Measured using a numerical rating scale, score range 0-10.

^b^BOA: Better Management of Patients With Osteoarthritis. Pain measured using a visual analog scale, score range 0-100.

^c^N/A: not available.

Investigation of each dimension of EQ-5D-3L separately showed that mobility and pain or discomfort were significantly improved from baseline to follow-up, while changes in self-care, usual activities, and anxiety or depression were not significant. Of participants who completed the 6-week program, 22.0% (55/250) reported that they had reduced the amount of OA-related medication, while 24.1% (13/54) no longer desired surgery (13 of 54 individuals altered their opinion).

The comparison between Joint Academy and the BOA initiative showed similar levels of pain at 3 months but suggested a greater reduction in Joint Academy due to a higher level of pain at baseline (Table 3).

## Discussion

### Principal Findings

The results for the 250 patients included in this study confirm in a larger cohort the previous preliminary findings of decreased pain attributed to participation in Joint Academy [12]. This study also extends knowledge by showing improved physical function for OA patients engaged in Web-based treatment for OA. Patients with OA commonly harbor doubts and fears of using their joints, which may erect a barrier to physical activity [19], a cornerstone treatment of OA. Although we used no validated instruments to assess walking difficulty and fear of movement (kinesiophobia), which may be considered a weakness in the methods, the results suggest that software as medicine may alleviate the patient’s concerns regarding being physically active due to pain and OA. Furthermore, despite the short intervention time in this study, 2 of the dimensions in the EQ-5D-3L scale improved (mobility and pain or discomfort). However, the index did not reach the change that is considered clinically significant (ie, a change of 0.074 [20]). Longer participation with continuous treatment would determine whether EQ-5D-3L scores would continue to improve. This study highlights the potential of a well-structured eHealth program to support patients in managing their symptoms and achieving minimal clinically important changes in chronic musculoskeletal conditions [21]. In addition, the fact that only a minority of all people with OA receive adequate treatment can be readily overcome because Web-based treatments such as Joint Academy are easily scalable.

According to recent systematic reviews analyzing Web-based treatment for musculoskeletal conditions in general and OA in particular, positive effects on physical function and pain were observed [10,11]. Similar to this study, in previous studies the changes in outcomes were, if not superior to, at least comparable with regular face-to-face treatment [7]. The comparison between Joint Academy and BOA on 3-month outcomes (6-week results are not available in BOA) serves as a preliminary report of results, and should be interpreted with caution, since it is based on 2 different sample populations and was not randomized. Such a comparison should ideally be evaluated in a randomized controlled trial, preferably combined with a health-economic analysis. In this respect, the finding that some patients changed their wish for surgery and need for painkillers after the 6-week program is noteworthy. A randomized controlled trial may also answer whether short, but regular, exercises performed daily using a Web-based treatment is more beneficial than outpatient visits twice a week for longer times. As shown here, patients need to spend 5 to 10 minutes every day to achieve significant improvement in only 6 weeks.

The strength of the program is its origin in evidence-based international guidelines for the primary management of knee and hip OA. Inherent in Web-based treatments is the possibility to individualize the treatment based on patient data, as well as being accessible around the clock, without demanding any equipment except for a smartphone or a computer. Furthermore, regular push emails and an encouraging physiotherapist available online as a support are most likely an advantage that should not be underestimated.

It is also important to recognize that eHealth is in its early developmental stages. To decrease health care costs without compromising patient satisfaction and outcome is a goal that is still relatively far away but is gradually getting closer with technical and analytical advances in the field. Joint Academy specifically could be improved with a further increase in individualization of the program. Building an outcome database of Joint Academy participants and subjecting such a database to artificial intelligence and neural network analysis would enable considering each individual’s clinical phenotype in order to optimize the program and its delivered exercises.

### Conclusion

The reported data suggest that participation in Joint Academy is associated with a clinically relevant decrease in pain and an increase in physical function and HRQoL, as well as a decreasing fear of physical activity. This innovative Web-based OA treatment is scalable, is population specific, and can reach a large number of individuals with impaired joints who have Internet access. The results, seemingly similar to those obtained with a face-to-face supported OA self-management program, have to be confirmed in a randomized controlled trial.

## Abbreviations

BOA: Better Management of Patients With Osteoarthritis
EQ-5D-3L: 3-level version of the EQ-5D
HRQoL: health-related quality of life
OA: osteoarthritis
